# Red blood cells differentiated in vitro using sequential liquid and semi-solid culture as a pre-clinical model

**DOI:** 10.1186/s40164-021-00244-z

**Published:** 2021-10-29

**Authors:** Matthew Cannon, Hannah Phillips, Sidney Smith, Shaneice Mitchell, Kristina Landes, Payal Desai, John Byrd, Rosa Lapalombella

**Affiliations:** 1 Division of Hematology, The Ohio State University, Columbus, OH USA; 2 College of Veterinary Medicine, The Ohio State University, Columbus, OH USA; 3 Division of Pharmaceutics, College of Pharmacy, The Ohio State University, Columbus, OH USA; 4 The Ohio State University, Room 455C, OSUCCC Building, 410 West 12th Avenue, Columbus, OH 43210 USA

## Abstract

The in vitro erythrocyte differentiation model remains a strong, clinically relevant tool to model erythroid development in normal and disease related hematopoiesis. This model also has application to developing therapeutics for diseases related to red blood cells such as sickle cell anemia where targeting increased expression of fetal hemoglobin has been a major emphasis. Since the original protocol’s publication in 2002, many groups have published modified methodologies to address issues in efficiency of maturation and terminal enucleation, as well as in scalability. While all reports have merit and show efficient enucleation, the methodologies used vary widely in technique and cytokine content. Additionally, despite the strengths in these methods, reproducibility of efficient differentiation to the point of differentiation is difficult. To address these limitations, we developed a streamlined process where total PBMCs are first primed using the original liquid culture expansion phase (published in 2002) before being differentiated with minimal input via standardized, commercially purchased semi-solid medium culture pre-supplemented with erythropoietin. Our data show this methodology to produce similar levels of CD235/CD71 positivity as previous methods but with enhanced CD235 solo positivity and evidence of enucleated cells in comparison with other widely used methods. Given the difficulty and wide variation in in vitro differentiation techniques, we present this methodology as a streamlined methodology for production of mature erythroid cells with minimal input using easily purchased reagents.

**Supplementary Information:**

The online version contains supplementary material available at 10.1186/s40164-021-00244-z.

## Main text

Since its original publication in 2002, the in vitro Red Blood Cells (RBC) differentiation protocol developed by Migliaccio et al. has been widely utilized by hematologists as way to model erythroid development and red blood cell disses in vitro [4, 5]. This two-stage liquid co-culture system has been a massive step forward for the field of blood cell research but even today it is still limited by efficiency of enucleation and yield of mature erythrocytes. Despite this, its advantages have led it to be among the most prominent model systems used and it should come as no surprise that many groups have since expanded and modified the existing protocols [1, 2, 6].

Most of these modifications leave the expansion phase relatively untouched. Almost all protocols utilize stem cell factor (SCF), interleukin 3 (IL-3), and erythropoietin (EPO) as necessary factors to promote self-renewal and erythropoiesis. Instead, researchers have modified the differentiation step of this protocol to achieve enucleation. The strategies used to achieve enucleation over the last decade have varied wildly. For example, Miharada et al. requires supplementation with human serum, D-mannitol, adenine, disodium hydrogen phosphate dodecahydrate and mifepristone [6]. Contrasting this supplementation heavy protocol, Giarratana et al. instead co-cultures cells with stroma before changing culture to contain no additional cytokines [2]. While these modifications show evidence of increased efficiency and enucleation, it can prove difficult to re-establish these protocols in new laboratories and slight variations in technique can drastically alter efficiency.

In our research efforts to develop drugs for sickle cell anemia, we first adapted the original two-stage protocol (Additional file 1: Figure S1a). Upon successful establishment, we next sought to extend the in vitro culture method by adapting previously discussed literature [2, 6]. Cells were cultured under protocols described in Miharada et al. and Giarratana et al. (now referred to as enucleation A or B) (Additional file 1: Figure S2). Analysis of enucleation A showed expansion of the early erythroblast population (CD235 + /CD71 +) but no substantial expansion of the late erythroblast population (CD235 + /CD71−) (Additional file 1: Figure S2b). Cells cultured through enucleation phase B produced CD235 + /CD71− late-stage erythroblast but saw a smaller erythroid population overall (Additional file 1: Figure S2b). No enucleation was detected via wright-giemsa staining in either condition. Our results suggest that while each methodology has merit in achieving erythroid differentiation, efficient maturation of this population is difficult to achieve.

**Fig. 1:**
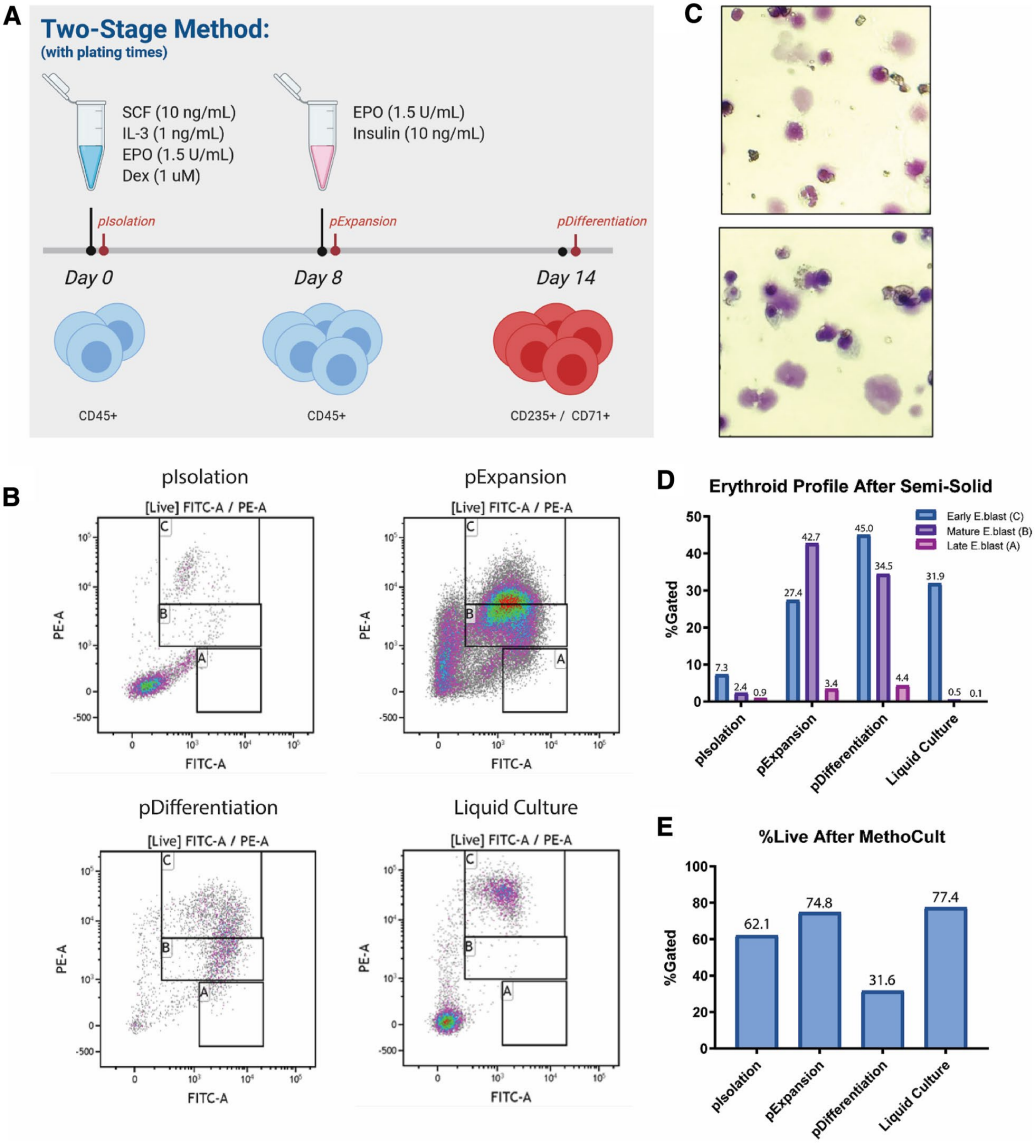
Sequential liquid culture and semi-solid media culture system produces robust erythroid response. **a** Timeline schematic of semi-solid medium plating schedule using the original two-stage methodology. Cell populations were plated in semi-solid medium containing EPO after each major end point: post-isolation, post-expansion, and post-differentiation. These are noted in red. **b** CD235/CD71 erythroid profiles of cells grown in semi-solid medium plated after major end points as indicated. **c** Wright-giemsa staining of enucleated erythroid cells grown in semi-solid medium (originally plated at post-expansion end point). **d** Quantification of CD235/CD71 erythroid profiles of semi-solid medium grown cells. **e** Quantification of live/dead staining of semi-solid medium grown cells

**Fig. 2:**
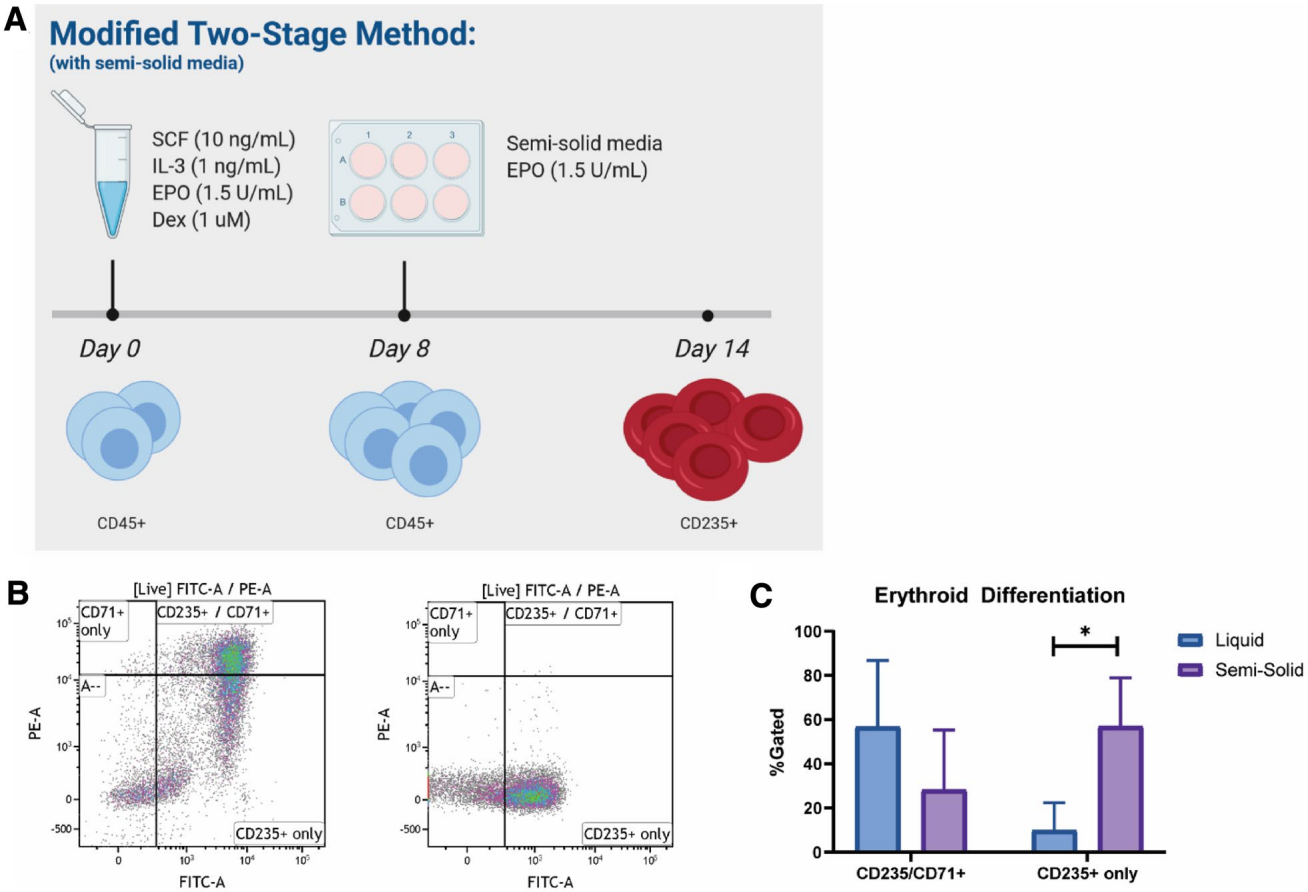
In vitro differentiated RBCs grown in sequential culture show high CD235 positivity. **a** Timeline schematic of modified two-stage methodology. At day 8, cells are collected and plated in semi-solid medium containing EPO for an additional week. **b** Sample flow diagram for erythroid profiles after sequential culture systems. Cells are gated on live populations. RBC flow control is shown on right panel. **c** CD235 positivity in erythroid profiles after sequential culture system or only liquid culture system (p  = 0.0117)

With these protocol modifications proving difficult to establish, we turned towards other potential methods. In the 1970s, the use of semi-solid media was found to be effective for growth of colony-forming units (CFU) [3, 7, 8]. Semi-solid media was further found to support the growth and expansion of erythroid cells through erythroid colony forming units (CFUe). Thus, we hypothesized that we could enhance the in vitro erythroid differentiation protocol by first using the liquid culture system to “prime” for erythroid colonies that could then be plated and expanded in standardized, commercially available semi-solid media supplemented with EPO. To test this, we repeated the original two-stage in vitro differentiation protocol and plated cells from each major timepoint in commercially available semi-solid media pre-supplemented with EPO. These major timepoints are outlined in Fig. 1a and are denoted in red. Robust erythroid differentiation was observed when cells were plated after the expansion phase of the original culture system (pExpansion) (Fig. 1b, d). Additionally, microscope inspection of wright-giemsa stained cells showed evidence of enucleation (Fig. 1c). These results led us to modify the original two-stage in vitro differentiation protocol slightly to include semi-solid media (Fig. 2a). After 1 week of culture in semi-solid media, cells grown in this manner showed CD235/CD71 positivity consistent with the original protocol, but with a larger CD235 + /CD71− population (Fig. 2b, c).

Interestingly, follow-up experiments revealed a “trade-off” effect between CD235 positivity and cell viability (Additional file 1: Figure S3). These results demonstrate that semi-solid medium can be used with the original expansion phase culture to produce robust erythroid differentiation.

From our experiments we show that standardized and commercially available semi-solid media supplemented with EPO can be utilized to easily create a robust differentiation and enucleation response. Given this, however, it should be also noted that while this method was effective in generating a differentiation response, due to the nature of semi-solid media, those seeking large scale expansion of differentiated red cells for larger scope applications would likely not benefit (Additional file 1: Figure S4). Instead, we suggest this method as an alternative methodology for those seeking to perform small-scale experiments on in vitro differentiated red cells in a pre-clinical setting.

## Supplementary Information

**Additional file 1: Figure S1. **Establishment of original two-stage culture methods.** a **Timeline schematic adapted from original in vitro erythroid culture system Migliaccio et al. [5]. Expansion phase cytokines are added after isolation. After 1 week, cells are cultured with differentiation phase cytokines. **b **CD45/CD235/CD71 positivity of patient derived PBMCs measured after 1 week in expansion phase cytokines (measured at day 8). **c **CD45/CD235/CD71 positivity of patient derived cells after 1 week in differentiation phase cytokines (measured at day 14). **d **Quantification of CD45/CD235/CD71 positivity at day 8 and day 14. **e **Quantification of differentiation status by CD235/CD71 status (Early E. blast: CD235^+^/CD71^+^, Mature E. blast: CD235^+^/CD71^dim^, Late E. blast: CD235^+^). **Figure S2.** Extension of two-stage culture methodology yields mixed results. **a **Timeline schematic for extension of two-stage culture system adapted from previously published methodology (Top-panel: Miharada et al. [6], Bottom-panel: Giarratana et al. [2]. **b **CD45/CD235 positivity for cells measured after final collection of both extended protocols. **Figure S3.** Semi-solid media demonstrates relationship between differentiation efficiency and cell viability. **a **CD235/CD71 erythroid profiles of semi-solid plated cells at various concentrations. **b **Quantification of CD235/CD71 erythroid profiles of various concentrations in semi-solid medium. **c **Viability of cells grown at various concentrations in semi-solid medium. **Figure S4. **Advantages and disadvantages at-a-glance between semi-solid and liquid culture for terminal stages of in vitro differentiation method.

## Data Availability

All data generated or analyzed during this study are included in this published article (and its Additional files). The datasets analyzed during this study are available from the corresponding author on reasonable request.

## Abbreviations

CFU: Colony-forming unit
EPO: Erythropoietin
IL-3: Interleukin-3
PBMC: Peripheral blood mononuclear cell
RBC: Red blood cell
SCF: Stem-cell factor
