# Supplementary figures and images for: Red blood cells differentiated in vitro using sequential liquid and semi-solid culture as a pre-clinical model
Source: Exp Hematol Oncol. 2021 Oct 29;10:50. doi: 10.1186/s40164-021-00244-z (PMC8555309; doi:10.1186/s40164-021-00244-z)

*
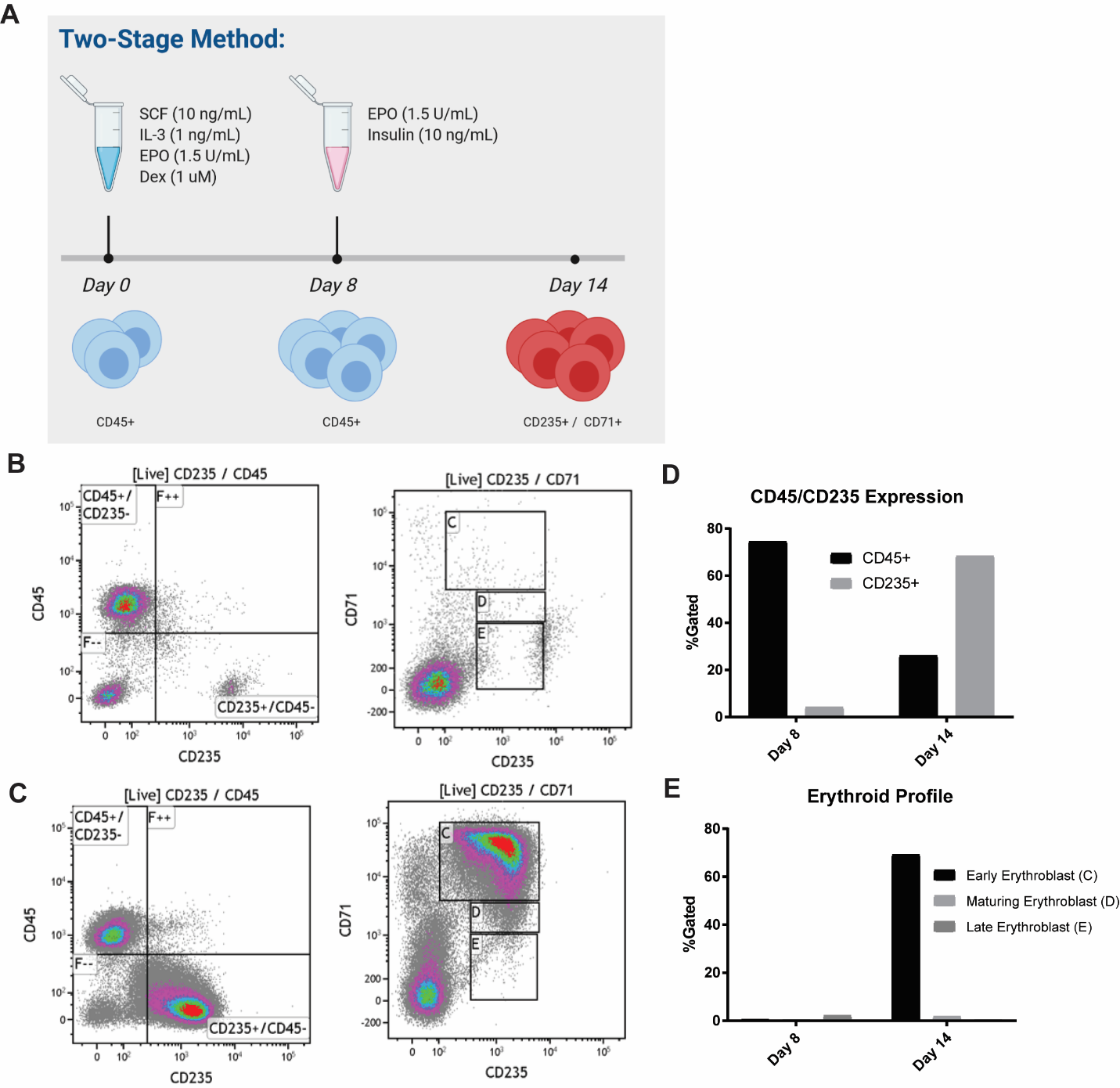
*

**Supplemental Figure 1**

**Supplemental Figure 2**

**Supplemental Figure 3.**


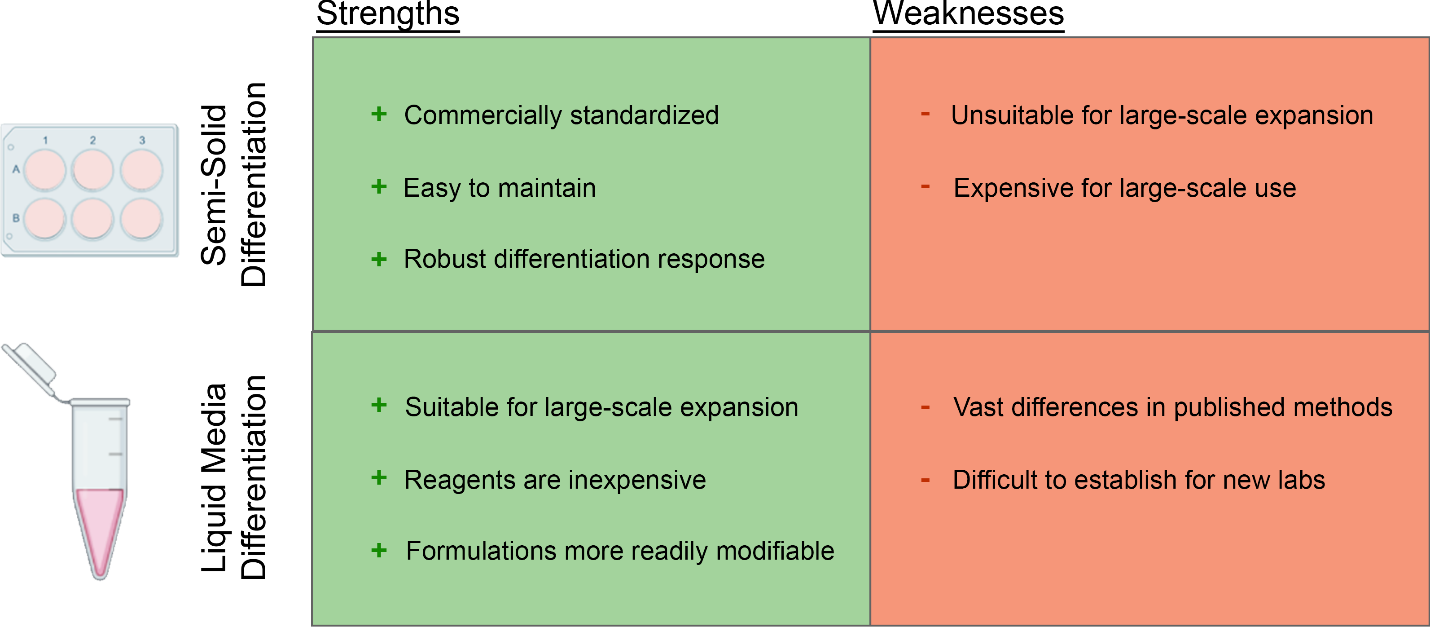


**Supplemental Figure 4**

Supplement: Supplementary file 1 — Additional file 1: Figure S1. Establishment of original two-stage culture methods. a Timeline schematic adapted from original in vitro erythroid culture system Migliaccio et al. [5]. Expansion phase cytokines are added after isolation. After 1 week, cells are cultured with differentiation phase cytokines. b CD45/CD235/CD71 positivity of patient derived PBMCs measured after 1 week in expansion phase cytokines (measured at day 8). c CD45/CD235/CD71 positivity of patient derived cells after 1 week in differentiation phase cytokines (measured at day 14). d Quantification of CD45/CD235/CD71 positivity at day 8 and day 14. e Quantification of differentiation status by CD235/CD71 status (Early E. blast: CD235+/CD71+, Mature E. blast: CD235+/CD71dim, Late E. blast: CD235+). Figure S2. Extension of two-stage culture methodology yields mixed results. a Timeline schematic for extension of two-stage culture system adapted from previously published methodology (Top-panel: Miharada et al. [6], Bottom-panel: Giarratana et al. [2]. b CD45/CD235 positivity for cells measured after final collection of both extended protocols. Figure S3. Semi-solid media demonstrates relationship between differentiation efficiency and cell viability. a CD235/CD71 erythroid profiles of semi-solid plated cells at various concentrations. b Quantification of CD235/CD71 erythroid profiles of various concentrations in semi-solid medium. c Viability of cells grown at various concentrations in semi-solid medium. Figure S4. Advantages and disadvantages at-a-glance between semi-solid and liquid culture for terminal stages of in vitro differentiation method. [file 40164_2021_244_MOESM1_ESM.docx]
